# Towards a Wearable Feminine Hygiene Platform for Detection of Invasive Fungal Pathogens via Gold Nanoparticle Aggregation

**DOI:** 10.3390/mi15070899

**Published:** 2024-07-10

**Authors:** Kimberley Clack, Mohamed Sallam, Carney Matheson, Serge Muyldermans, Nam-Trung Nguyen

**Affiliations:** 1 Queensland Micro and Nanotechnology Centre (QMNC), Griffith University, Nathan Campus, Nathan, QLD 4111, Australia; kimberley.clack@griffithuni.edu.au (K.C.); mohamed.sallam@griffithuni.edu.au (M.S.); 2 School of Environment and Science (ESC), Griffith University, Nathan Campus, Nathan, QLD 4111, Australia; c.matheson@griffith.edu.au; 3 Griffith Institute for Drug Discovery (GRIDD), Griffith University, Nathan Campus, Nathan, QLD 4111, Australia; 4 Laboratory of Cellular and Molecular Immunology (CMIM), Vrije Universiteit Brussel, 1050 Brussels, Belgium; serge.muyldermans@vub.be

**Keywords:** women, gold nanoparticles, wearable, *Candida albicans*, colorimetric

## Abstract

*Candida albicans* is an opportunistic fungus that becomes pathogenic and problematic under certain biological conditions. *C. albicans* may cause painful and uncomfortable symptoms, as well as deaths in immunocompromised patients. Therefore, early detection of *C. albicans* is essential. However, conventional detection methods are costly, slow, and inaccessible to women in remote or developing areas. To address these concerns, we have developed a wearable and discrete naked-eye detectable colorimetric platform for *C. albicans* detection. With some modification, this platform is designed to be directly adhered to existing feminine hygiene pads. Our platform is rapid, inexpensive, user-friendly, and disposable and only requires three steps: (i) the addition of vaginal fluid onto sample pads; (ii) the addition of gold nanoparticle gel and running buffer, and (iii) naked eye detection. Our platform is underpinned by selective thiolated aptamer-based recognition of 1,3-β-D glucan molecules—a hallmark of *C. albicans* cell walls. In the absence of *C. albicans*, wearable sample pads turn bright pink. In the presence of *C. albicans*, the wearable pads turn dark blue due to significant nanoparticle target-induced aggregation. We demonstrate naked-eye colorimetric detection of 4.4 × 10^6^ *C. albicans* cells per ml and nanoparticle stability over a pH range of 3.0–8.0. We believe that this proof-of-concept platform has the potential to have a significant impact on women’s health globally.

## 1. Introduction

*Candida albicans* (*C. albicans*) is a commensal fungus that colonizes the intestinal microbiota in most of the human population [1]. However, an imbalance of the microbial flora, dysfunction of the immune system, or breakage of epithelial barriers favors the transition from commensal fungus to an invasive pathogen [2]. *C. albicans* is the most common human pathogenic candida species, and it can cause a range of diseases, including mucosal, skin, and systemic infections (candidiasis) [2]. *C. albicans* is the leading cause of candida bloodstream infections (candidemia). Candidemia is associated with significant hospital costs and may even cause deaths in immunocompromised patients [3]. Vulvovaginal candidiasis (VVC) is exceptionally common, and it impacts 3 in 4 women at least once during their lifetime [4]. Five percent of women develop recurrent vulvovaginal candidiasis (RVVC) [5]. Vulvovaginal infections (VVI), such as VVC, may cause vaginal symptoms such as itching, odor, soreness, dyspareunia, and vaginal discharge [4,5]. These symptoms also include larger social and psychological consequences [4]. If left untreated, recurrent VVI may lead to complications such as pre-term birth, infertility, miscarriages, and other infectious diseases such as human immunodeficiency virus (HIV) [5]. Due to these adverse effects on women’s reproductive health and well-being, vaginal infections are a major health concern globally. VVC risk factors include pregnancy, reproductive age, hormone replacement therapy, immunosuppression, antibiotic use, receptive oral sex, uncontrolled diabetes, oral contraceptive pills, and frequent sexual intercourse [5]. Therefore, early and accurate diagnosis of VVC is an essential pathway for improving patient health and quality of life and for reducing deaths. Clinical diagnosis of VVC may be performed by saline and potassium hydroxide microscopy testing; however, the sensitivity of this method is low, even in experienced hands [6]. When VVC is still suspected despite a negative microscopy result, a fungal culture should be obtained. This sample may be cultured on Sabouraud’s media to observe colony growth. This method is still the “gold standard” method for VVC diagnosis; however, it is expensive and may take up to 7 days to generate a result [7]. Polymerase chain reaction (PCR) methods may be used to detect candida genus presence as well as to determine the presence of a specific species.

This testing procedure is provided by commercial companies, and it provides reliable results within several days [6]. PCR testing is more sensitive than visible fungal culture analysis; however, PCR is more expensive and has not demonstrated any advantage to the clinician in practice [6]. Additionally, these methods are worthless if the patient fails to consult their general practitioner. As more over-the-counter treatments are becoming available, more women are self-diagnosing and self-treating for VVC [7]. However, misdiagnosis is common due to difficulty in distinguishing between bacterial vaginosis, trichomoniasis and yeast infection from symptoms alone. As many as two-thirds of over-the-counter drugs that are sold to treat VVC were used by women without the disease [7]. Overuse of antifungal medications is problematic due to fungal resistance to these medications. Therefore, it is imperative to create a simple, rapid, and user-friendly platform for VVC diagnosis for patients to use at home. This platform would significantly reduce healthcare costs and would lead to better management of VVC overall [7]. Glucans are the most abundant polysaccharides in fungal cell walls, and they offer an alternative detection approach to conventional detection methods [8] β-1,3-d-glucan (BDG) molecules comprise 84% of fungal glucan molecules [8]. Currently, the Fungitell platform (Cape Cod, MA, USA) is the only Food and Drug Administration (FDA) approved BDG detection platform. However, it requires serum, and it may be prone to false-positive results [9]. Colorimetric detection platforms offer a simple and practical way to indicate the presence of a target analyte or biomolecule such as BDG. Colorimetric platforms may circumvent the need for complex data generation, instead, offering a naked-eye detectable result [10]. Recent colorimetric fungal detection platforms have included (but are not limited to) loop-mediated isothermal amplification (LAMP) for detecting the presence of amplified target fungal DNA [11,12]; HRP-mediated TMB oxidation (horseradish peroxidase/Tetramethylbenzidine) assay [13] and surface plasmon resonance-based techniques [14]. However, these techniques suffer from the need for expensive and complex equipment or laboratory facilities. Gold nanoparticles are well-suited for colorimetric sensing due to their surface plasmon resonance-enhanced optical properties. They also include other features such as controllable size, well-established surface chemistry, facile and well-established synthesis and catalytic activity [15]. However, we previously reported on [16] some of the limitations associated with emerging gold nanoparticle colorimetric platforms, which include, but are not limited to, the requirement for expensive and specialized equipment. To address these needs, we have expanded on our prior work [16] to create a potentially wearable, rapid, naked-eye-based colorimetric method for *C. albicans* BDG detection via thiolated (3′ end) aptamer-conjugated gold nanoparticles. Our new platform offers detection of *C. albicans* directly from wearable substrates that are designed to be adhered to existing sanitary pads, that are worn by women. Our platform is designed to collect vaginal fluid samples without the need for the patient to insert a cotton swab into or around the vagina. This substrate-based method of vaginal fluid collection is minimally invasive, and it decreases the likelihood that the patient may contaminate the sample or their surroundings. Our method offers a proof-of-concept, easily perceptible “blue” color change for a positive result and a “pink” color for a negative result. To the best of our knowledge, no wearable colorimetric aptamer-gold-nanoparticle-based platform exists for the direct detection of *C. albicans* from vaginal fluid. Additionally, our platform could detect *Candida albicans* from both wet and dry samples. The platform was also stable over a wide pH range (3.0–8.0) and was not prone to nonspecific aggregation within a complex biological media (human plasma). The tested cell concentration (that could still give a bright distinction between positive and negative tests) was as low as 4.4 × 10^6^ cells per ml from a sample volume of 100 μL. We believe that this detection value is clinically relevant as at any given time, 0.5 to 0.75 g or 0.5 to 0.75 mL of vaginal fluid is present within a patient sample [17]. Additionally, we believe that our results are within the range of clinical feasibility concerning the available number of *C. albicans* cells that are present within symptomatic vulvovaginal candidiasis cases (10^6^ cells per sample of vaginal fluid) [18]. However, we will improve upon this proof-of-concept detection limit with further optimization of future work.

## 2. Materials and Methods

### 2.1. Vaginal Fluid Simulant and Platform Stability Testing

Vaginal fluid is mainly comprised of proteins, salts, fatty acids, and carbohydrates. For clinical relevance, our platform was tested within a vaginal fluid simulant solution across all experiments. For our work, a vaginal fluid simulant solution was prepared from the recipe used by Owen and Katz (1999) [17]. The recipe consisted of NaCl (MW. 58.4), KOH (MW. 56.1), Ca(OH)_2_ (MW. 74.1), Bovine Serum Albumin, Lactic acid (L+) (MW. 90.1), glacial acetic acid (MW. 60.1), glycerol (MW. 92.1), urea (MW. 60.1) and D-glucose monohydrate (MW. 198.2). The combined reagents were diluted to a total reaction volume of 1 L with Milli-Q water. The solution was adjusted to pH 4.2 with HCl and was vacuum filtered and UV sterilized before being aliquoted for later use. This pH adjustment is in concordance with the pH of the average healthy vagina being around 4.0 ± 0.5. However, this pH value differs across ethnicity and geographical locations [19].

For platform stability testing, the stock solution of vaginal fluid solution (pH 4.2) was aliquoted, and the pH was adjusted to create various solutions ranging from pH 2.0 to pH 8.0. pH was adjusted up or down with 1.0 M NaOH or 1.0 M HCl, respectively. Additionally, human plasma in 4% trisodium citrate (Sigma Aldrich, St. Louis, MO, USA) was used without dilution for testing platform stability in a complex biological setting. Unless specified, all experimental reagents were purchased from Sigma Aldrich, St. Louis, MO, USA.

### 2.2. Fungal Culture, Preparation and Fungal Cell Count Estimation

*Candida albicans* were cultured on yeast extract peptone dextrose (YEPD) plates that were created according to the following recipe: 5 g of yeast was dissolved in 250 mL of MilliQ water. 10 g of peptone (MW. 244.33) was added, as well as 10 g of glucose (MW. 180.2), followed by 10 g of agar. The solution was heated to combine the reagents, and MilliQ water was added to the 500 mL mark. The solution was autoclaved and stored at 4 °C overnight, ready for plate pouring the following day. *C. albicans* strains were cultured by adding a portion of lyophilized stock (*Candida albicans* (Robin) Berkhout—ATCC 24433) to 400 μL of Miller′s LB broth solution and covering YEPD plates with the fungal solution to ensure colony distribution. Plates were incubated at 28 °C. After five days of growth, fungal colonies were harvested for experiments.

*Botrytis Cinerea* was cultured on V8 agar plates that were created according to the following recipe: 8 g of bacteriological agar, 1.5 g of CaCO_3_ (MW. 100.1) (previously dissolved in 50 mL of MilliQ water), and 90 mL of V8 vegetable juice (Campbell Soup Company, Camden, NJ, USA) were combined, and MilliQ water was added to the 400 mL mark. The solution was vacuum filtered, autoclaved and stored overnight (at 4 °C) and was available for heating and plate pouring the following day. *Botrytis cinerea* was obtained in-house and was cultured by slicing a portion of agar from the stock fungal plate and placing it onto a fresh plate incubated at room temperature. Spores were harvested by scrubbing the plates with pipette tips after five days of growth. Due to the nature of the extensive mycelium of the *Botrytis cinerea* fungus, these cells were not quantified for our work. Instead, an excess of this fungus was used for assay specificity testing as per our prior method [16].

*C. albicans* fungal cell counts were estimated using optical density readings. At OD_600_ = 0.5, an approximate fungal cell concentration of 1 × 10^7^ cells/mL is expected [20]. For our serial dilution analysis, *Candida albicans* cells were harvested from cultures and were spiked into a volume of vaginal fluid simulant solution. Optical density was measured by diluting the stock fungal cell solution, and it was determined to be 0.35 (average of three readings). It was determined that the stock solution had an abundance of *Candida albicans* cells (700 million cells per 1 mL). From this stock, 1:1 serial dilutions in vaginal fluid simulant solution were performed in preparation for data generation for subsequent testing.

### 2.3. Wearable Substrate Assembly

Various substrates were tested for suitability for the wearable platform. These included gauze swabs (7.5 cm × 7.5 cm—8 ply, BSN Medical GmbH, Hamburg, Germany); chromatography paper (1 CHR 20 cm × 20 cm—Whatman Schleicher & Schuell, Munich, Germany); cellulose fiber sample pads (20 cm × 30 cm-EMD Millipore Corporation, Burlington, MA, USA), Libra sanitary pads (Melbourne, Australia) and glass fiber diagnostic pads (GFDX203000) (EMD Millipore Corporation, Burlington, MA, USA). After initial substrate testing, all subsequent experiments were performed with the glass fiber pad due to its performance, as reported in the discussion section. The glass fiber pad was assembled into a final wearable platform consisting of two parts: The glass fiber diagnostic pad and the polystyrene backing card (FF170HP) (Whatman, GE Healthcare Life Sciences, Buckinghamshire, UK). The polystyrene backing card was incorporated to give rigidity and stability to the glass fiber diagnostic pads so that they could be easily handled for image analysis. Initially, both the glass fiber diagnostic pad and polystyrene backing cards were cut to 1.5 cm × 1.5 cm dimensions each. The glass fiber diagnostic pads were then adhered to the polystyrene backing cards after removing the polystyrene backing card adhesive strips. With some modification, these backing cards can be made to be adhesive (to the sanitary napkins) and compatible with the skin. This requires adding a gluing agent to the back of the cards and a thin layer of gauze or another suitable material to protect the skin from direct contact with the glass fiber pads. Other protective layers will be investigated to ensure that the skin is protected from the glass fiber layer while allowing samples of vaginal fluid containing fungal cells to still be deposited on the glass fiber layer. However, this is outside of the scope of this current proof-of-concept work.

### 2.4. Aptamer Design and Preparation

The AD1 aptamer (k_d_ = 79.76 nM) was selected for conjugation (via thiol linkage) to gold nanoparticles for specific binding to *Candida albicans* β-1,3-D-glucan molecules. AD1 can assist in detecting different morphological forms of *Candida albicans*, including yeast cells, germ tubes, and hyphae, as well as extracellular matrix material [8]. Our aptamer design is as follows:

5′GCGGAATTCGAACAGTCCGAGCCCACACGTGTGAGAAGGGTGTTATCATGTATTTCGTGTTCCTTTCGTCATTCCTTTGTCTGGGGTCAATGCGTCATAGGATCCCGCAAAAAAAAAA-3′Thiol Modifier C3 S-S. This aptamer was selected based on prior work from Tang et al. [21]. Most fungi contain glucans which may lead to potential false positive results in a BDG detection assay. To improve BDG detection specificity, Tang et al. created two aptamers (AD1 and AU1) that can specifically bind to two different domains of (1-3)-β-D-glucans that are highly specific for *Candida albicans* BDG only. They demonstrated high sensitivity and high specificity (92.31% and 91.94%, respectively) for detecting (1-3)-β-D-glucans in serum samples from different groups of patients with *Candida albicans* infection via a double-aptamer sandwich enzyme-linked oligonucleotide assay.

This lyophilized aptamer (Integrated DNA Technologies, Coralville, IA, USA) was reconstituted with RNAse-free water to form a 10 μM stock solution and was aliquoted for future use. Prior to nanoparticle synthesis, 2 μL of 10 μM aptamer solution was added to 250 μL of 0.01% Tween-20 solution to form the aptamer solution (for combination with gold nanoparticles) as described in the following section.

### 2.5. Nanoparticle Synthesis

To maintain nanoparticle purity during synthesis, a glass vial was rinsed in aqua regia (HCl, HNO_3_, 3:1), washed in distilled water, and air-dried. Aptamer-conjugated gold nanoparticles were created within this vial according to the following recipe: 440 μL of 0.1% Tween-20, 120 μL of 10 mM HAuCl_4_, 250 μL of 0.1 M sucrose and 250 μL of AD1 aptamer solution were added to a glass vial under magnetic stirring (700 RPM at room temperature) for 5 min. Then, 25 μL of 1.0 M NaOH was added to the solution under magnetic stirring. The solution was stirred further for another ten minutes to ensure the complete formation of the gold nanoparticles. Upon nanoparticle formation, the solution turned a deep “red” color. This solution was then centrifuged (room temperature, 12,000 RPM) for 40 min, and the supernatant was removed and discarded. Nanoparticle pellets were obtained and added to a 0.5% agarose gel solution (1:2; nanoparticle: gel) via thorough pipette mixing. In general, nanoparticles may continue to grow for 24 h after synthesis, and those with less stable capping agents may continue to grow slowly after 24 h [22]. To ensure reproducibility and smaller size, our nanoparticles were combined with the gel within an hour of nanoparticle synthesis.

### 2.6. Running Buffer Preparation

Seven buffers were prepared for analysis according to existing literature: Sodium dodecyl sulfate detergent buffer (0.05%, pH 7.0) (SDS) [23]; buffer containing 20 mM sodium borate (pH 8.0), 2% (*w*/*v*), sucrose, 0.6 M NaCl, 0.2% (*v*/*v*) Tween 20%, and 0.1% (*w*/*v*) sodium azide [24]; high salt buffer (HSB: 0.1 M tris-HCl (pH 8.0), 0.01 M MgCl_2_, and 0.15 M NaCl) containing 30% (*v*/*v*) ethanol [25]; phosphate buffer (0.01 M, pH 7.4)—made by dissolving 0.21 g of Na_2_HPO_4_ in 150 mL of Milli-Q water, and adjusting to pH 7.4 with NaH_2_PO_4_·H_2_O solution (made by dissolving 0.20 g of NaH_2_PO_4_·H_2_O in 150 mL of Milli-Q) [23]; tris buffer (pH 7.5, 1.0 M) [23]; HEPES buffer (pH 7.4, 10 mM) [23] and finally a buffer comprised of 20 mM sodium borate (pH 8.0), 0.8 M potassium chloride and 0.2% *v*/*v* Tween-20 solution from the recipe from Park and colleagues [24] Due to the performance of the final buffer (as reported within the discussion), this buffer was used for all experiments after initial buffer testing.

### 2.7. Image Processing and Analysis

The images presented in this study were photographed with an iPhone 15 pro. All images were cropped to the same size (220 × 220 pixels) and were then imported into ImageJ 1.5.4g software (Java 1.8.0_345 64-bit, National Institutes of Health, Madison, WI, USA) for analysis. Subsequently, the background color of all the images was subtracted (with the light background checkbox selected). Then, each of the images was converted into red, blue, and green channel individual images by splitting the color channels. The red channels were subsequently selected and were measured via ImageJ to give a final numerical value for the brightness intensity. Red channel analysis was ideal because as gold nanoparticle aggregation increases, so does the “blue” color, and therefore, the brightness intensity of red pixels decreases. Red channel brightness intensity values were then entered into OriginLab graphing software (version: 10.1.0.178) (Northampton, MA, USA) for graphical representation. Error bars are displayed where appropriate for *n* = 3 sample replicates. Error bars are not displayed when results are a single value representative of an average of the three replicates.

## 3. Results and Discussion

### 3.1. Assay Principle

**Figure 1:**
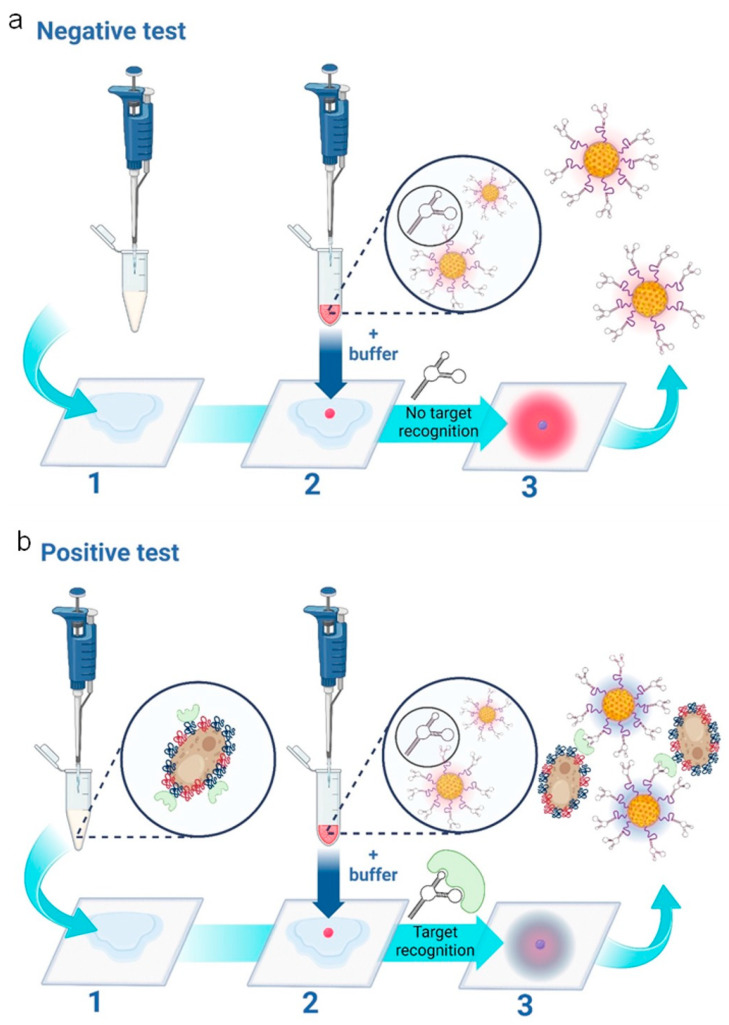
Schematic representation of the three-step wearable platform. (**a**) Indicates a negative test for *C. albicans*. In step (1), 100 μL of vaginal fluid solution is added to the sample pad. (2) 5 μL of nanoparticle gel solution is added, as well as 45 μL of running buffer solution. A negative result is indicated in (3), showing a “pink/red” color due to the non-aggregation of the aptamer-conjugated gold nanoparticles. (**b**) indicates a positive test for *C. albicans*. In step (1), 100 μL of vaginal fluid solution containing *C. albicans* fungal cells is added to the sample pad. (2) 5 μL of nanoparticle gel solution is added, as well as 45 μL of running buffer solution. A positive result is indicated in (3), showing a “purple/dark blue” color due to the specific target-induced aggregation of the aptamer-conjugated gold nanoparticles. The scheme is illustrated and reproduced with permission from BioRender.

The assay principle is schematically presented in Figure 1, depicting the cases of both negative (Figure 1a) and positive tests (Figure 1b), respectively, for *Candida albicans*. The assay features three main steps: 1—Addition of vaginal fluid samples to wearable sample pads; 2—addition of the nanoparticle gel to the sample pad, followed by the addition of the running buffer and 3—a colorimetric result based on the aggregation state of the nanoparticles. In the case of a negative test result (the absence of *Candida albicans*), the sample pad turns bright pink, as the red nanoparticles remain free of aggregation. In the case of a positive test result (the presence of *Candida albicans*), the sample pad turns dark blue. This is caused by aptamer-target-induced nanoparticle aggregation, i.e., nanoparticle aggregation results in a redshift (or blue color change) on the plasmonic spectrum [26].

Lateral flow platforms (LFAs) are considered to be simple, stable, and suitable for various environments, such as resource-limited regions. However, lateral flow platforms are also associated with limited detection range, batch-to-batch variation, and long development time due to the required animal ethics approval [27,28]. Additionally, traditional antibody-based lateral flow platforms may require specialized cooling conditions or careful handling, adding to transportation costs [29]. Incorporating surface-enhanced Raman scattering (SERS) technology into lateral flow platforms expands their application range and greatly enhances their sensitivity [27]. A SERS-based lateral flow platform was developed to detect bacteria cells with high sensitivity (10^2^ and 10 cells/mL), using a combination of colorimetric and SERS detection methods, respectively [30]. However, in achieving this high level of sensitivity, the platform required manual handling, and sample enrichment by a magnetic field. Magnetic enrichment may require a skilled user and may not be unsuitable for a home setting. Another SERS-based lateral flow platform was developed for bacterial detection [31]. Platform sensitivity was 27 CFU/mL for *S. Enteritidis* and 19 CFU/mL for *L. monocytogenes*. However, a confocal micro-Raman spectroscopic system was required for signal generation. This equipment is expensive and may not be suitable for on-site detection [32]. Additionally, the requirement for lasers and interpretation of SERS signals presents an additional barrier to manufacturing a portable, wearable, and cost-effective platform, especially within resource-limited settings.

Typical lateral flow platforms utilize the glass fiber diagnostic pad as the platform’s conjugate pad. The conjugate pad has three main functions: preserving dried nanoparticles, releasing them upon sample wetting, and providing the first interaction between the target and labeled bioreceptor [23]. Typically, conjugate pads of lateral flow assays incorporate buffering agents to maximize nanoparticle stability and to completely release them upon rewetting by the sample [23]. Once the conjugate pad buffer has been chosen, the nanoparticle conjugates are loaded onto the conjugate pad by either immersion or air jet dispensing. Air jet dispensing requires a costly and well-calibrated dispensing apparatus, and the immersion method may have non-uniform coverage, leading to sensor-to-sensor variability [23]. Furthermore, within LFA construction, the conjugate pad requires drying in hot air (which is typically fixed at 37 °C) or vacuum drying. Drying is critical for maintaining the stability of the dried nanoparticle-bioreceptor conjugates [23]. Improper conjugate pad drying can greatly impact the nanoparticle release efficiency from the conjugate pad. Overall, this leads to batch-to-batch variability and reduced platform sensitivity. Much like the LFA, our method is disposable, cheap, rapid, portable, and convenient. However, our method avoids the need for nitrocellulose test strips, substrate pretreatment, or careful and precise biomolecular stripping or nanoparticle/bioreceptor functionalization onto conjugate pads. We believe that this greatly improves batch-to-batch stability due to the simplicity of the substrate of our platform and reduces substrate manufacturing time. Furthermore, the wearable substrate is not subject to strict storage conditions such as cooling or careful packing or handling, and it is functional at room temperature, eliminating the need for expensive temperature control equipment. We believe that our platform has significant clinical translational potential because the wearable substrate can be adhered directly to existing sanitary napkins or underwear (with some modification) for testing with a user-friendly nanoparticle gel-based solution and buffer.

### 3.2. Initial Concept and Running Buffer Selection

The type and concentration of running buffer can greatly influence overall pH and ionic strength. Ionic strength and pH can affect the interaction between the receptor and target as well as affect potential nonspecific binding. This can greatly impact the sensitivity, specificity, and reproducibility of a biomolecular detection platform. The performance of various running buffers was assessed within our platform to determine which buffer would show the best distinction between positive and negative samples. Running buffers were selected for our platform based on commonly reported buffers within LFA platforms. LFA buffers were selected due to their performance in enabling the efficient release of the nanoparticles from the middle of the sample pads to easily visualize colorimetric results.

**Figure 2:**
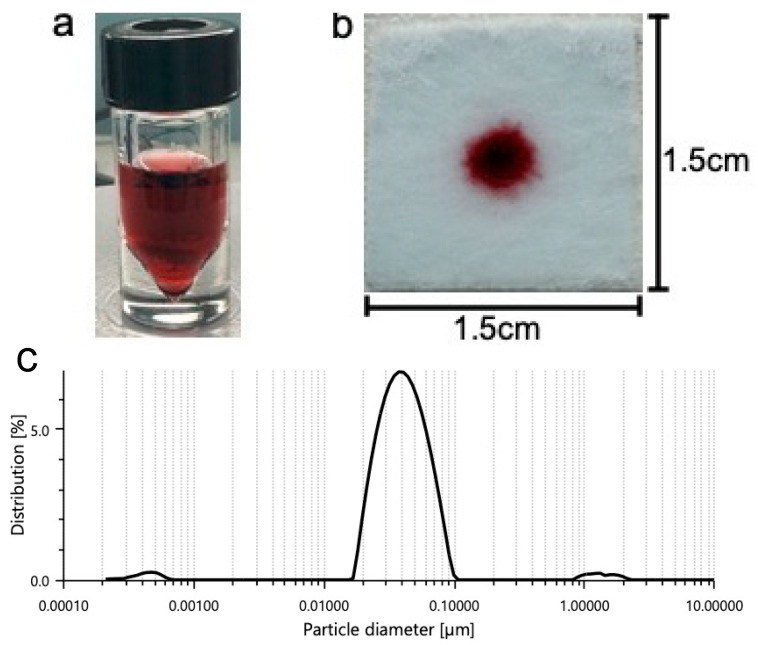
A solution of gold nanoparticles prior to centrifugation and prior to combination with the agarose gel (**a**,**b**) 5 μL of nanoparticle gel solution is dispensed onto the substrate containing 100 μL of previously soaked vaginal fluid simulant solution prior to running buffer addition. Prior to the running buffer, the nanoparticles remain confined to the gel matrix and do not disperse across the substrate. (**c**) Shows one prominent peak indicating a mean particle hydrodynamic diameter of 0.04 μm.

Some of these buffers include phosphate buffer (pH range between 5.8 and 8.0), tris buffer (pH range between 7.5 and 9.0), and HEPES buffer (pH range between 6.8 and 8.2). Common detergents such as SDS are typically used within the sample pad portion of LFA platforms. Detergents may assist with minimizing nonspecific binding (disrupting weak ionic and hydrophobic bonds) and with facilitating the flow of the detection labels along the different pads. Consequently, we tested SDS to observe its effect on our platform. Colloidal nanoparticle suspension stability is generally affected by the ionic strength of the solution. As such, buffers containing borate may be used due to their low ionic strength. Figure 2a shows the prepared gold nanoparticles prior to centrifugation and prior to addition to the gel. Figure 2b shows 5 μL of nanoparticle gel solution that is dispensed onto the substrate containing 100 μL of previously soaked vaginal fluid simulant solution prior to running buffer addition. Prior to the running buffer, the nanoparticles remain confined to the gel matrix and do not disperse across the substrate. We believe that incorporating the nanoparticles into a gel, greatly improves manual handling, allowing for more uniform distribution of nanoparticles by the user.

Dynamic light scattering (DLS, LiteSizer 500, Anton Paar GmbH, Graz, Austria) was used to evaluate the size distribution of the prepared nanoparticles prior to combination with the gel. The DLS data show a mean particle hydrodynamic diameter of 0.04 μm (Figure 2c). DLS revealed a mean polydispersity index of 20.4%, indicating that the nanoparticles are monodispersed and potentially stable before they are used for experimentation.

**Figure 3:**
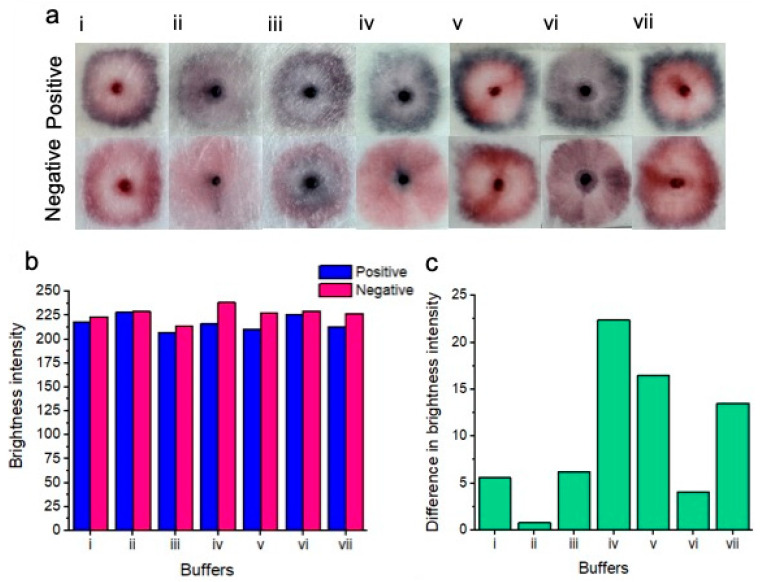
Colorimetric results of buffer testing (**a**). Positive tests for each buffer are shown on top, and negative tests are shown on the bottom. From left to right, the buffers include SDS (i); sodium borate/sucrose/NaCl/Tween/Sodium azide (ii); tris-HCl/MgCl_2_/NaCl and ethanol (iii); sodium borate/KCl/Tween (the buffer chosen for subsequent experimentation) (iv); phosphate buffer (v); tris-HCL buffer (vi) and HEPES buffer (vii). Only buffers (iv), (v), and (vii) showed a naked-eye distinction between positive and negative samples. The remaining buffers did not show an easily perceptible distinction between positive and negative results based on subjective naked-eye interpretation. Red channel brightness intensity for positive and negative samples after image processing is shown (**b**). Difference between red channel brightness intensity for positive and negative results for each of the tested buffers. (**c**) shows that buffer iv gives the best naked-eye detectable distinction between positive and negative samples.

The results of various buffer tests are displayed in Figure 3. A concentration of 7.0 × 10^7^ *Candida albicans* cells per mL was tested to ensure the best distinction could be made between positive and negative tests for each tested buffer. Results of buffer testing are described as follows, describing the outer portion, inner portion and nanoparticle gel conditions (in terms of naked-eye clarity) of each of the tests.

SDS buffer (i)—the distinction was unclear between positive and negative samples. Positive samples showed a pattern of nanoparticle aggregation in the form of a dark pink outer ring, whereas the center of the positive test showed a pink unaggregated portion as well as a pink coloration of the nanoparticle gel. Negative tests showed a bright pink outer ring, a pink inner color as well as a pink coloration of the nanoparticle gel.

Sodium borate/sucrose/NaCl/Tween/Sodium azide buffer (ii)—the distinction was unclear between positive and negative samples. Positive samples showed a slight pattern of nanoparticle aggregation in the form of a thin dark/purple outer ring. The center of the test showed slightly aggregated purple nanoparticles as well as a dark purple color of aggregated nanoparticles within the nanoparticle gel. The negative test showed no definitive nanoparticle ring, however, the inner portion of the test was a pale pink color. The nanoparticle gel of the negative test resembled the same aggregated purple color as seen within the positive test.

Tris-HCl/MgCl_2_/NaCl and ethanol buffer (iii)—the distinction was unclear between positive and negative tests. Positive samples showed a purple outer ring of aggregated nanoparticles, while the inner portion of the test showed a feint, aggregated dark blue color. The nanoparticle gel of the positive test showed a dark blue to black color of aggregated nanoparticles. The negative test showed a pink outer ring of unaggregated nanoparticles, whereas the center of the test showed significant non-specific nanoparticle aggregation. The nanoparticle gel of the negative test showed a dark blue to black color of aggregated nanoparticles.

Sodium borate/KCl/Tween buffer (iv)—a very clear distinction between positive and negative samples was observed. Positive samples showed a dark purple to blue outer ring of aggregated nanoparticles. The inner portion of the test displayed a slightly aggregated, purple nanoparticle color, whereas the nanoparticle gel showed a dark purple to black color of aggregated nanoparticles. The negative test did not show a ring of nanoparticles. However, the inner portion of the test showed a relatively homogeneous portion of pink unaggregated nanoparticles. However, the nanoparticle gel of the negative test displayed a dark purple to blue color, suggesting some nonspecific nanoparticle aggregation.

Phosphate buffer (v)—a distinction was evident between the positive and negative tests. Positive samples showed a dark purple to blue outer ring of aggregated nanoparticles. The inner portion of the test showed a bright pink portion of unaggregated nanoparticles, however. The nanoparticle gel remained free of aggregated nanoparticles, showing a bright pink to red color. The negative test showed a dark pink color of nanoparticles of “borderline” aggregation status. The inner portion of the test showed dispersed nanoparticles that remained free of aggregation. As with the positive test, the nanoparticle gel remained free of visible aggregation and was pink to red in color.

Tris-HCL buffer (vi)—no clear distinction between positive and negative tests was observed. Positive samples showed a dark blue ring of aggregated nanoparticles, while the middle portion of the test showed slightly aggregated purple nanoparticles. The nanoparticle gel showed an aggregated dark purple color. The negative test did not display an aggregated ring portion of nanoparticles; however, the inner portion of the test showed some non-specific nanoparticle aggregation in the form of a purple-to-pink color. The nanoparticle gel showed nanoparticle aggregation in the form of a dark purple color.

HEPES buffer (vii)—a distinction was observed between positive and negative tests. Positive samples showed a distinct dark blue color of aggregated nanoparticles as an outer ring, whereas the inner portion of the test showed zero nanoparticle aggregation in the form of a bright pink color. The nanoparticle gel remained unaggregated in the form of a bright pink to red color. In the case of the negative test, the outer portion of the test displayed a dark pink color, possibly indicating potentially and slightly aggregated nanoparticles. The inner portion of the test remained aggregation-free and pink. The nanoparticle gel also remained unaggregated and bright pink to red in color.

Figure 3b displays the red channel brightness intensity of each buffer for the positive and negative samples after image processing. Notably, all buffers displayed a lower red channel brightness intensity for the positive tests compared to the negative tests. (Figure 3c) Shows the difference in brightness intensity (after image processing) between positive and negative samples for each of the buffers. Notably, buffer (iv) (sodium borate/KCl/Tween-20) showed the greatest difference in red channel brightness intensity between positive and negative results, i.e., the positive result was “blue”, which was low in red channel brightness intensity and the negative result was “pink”, which was high in red channel brightness intensity.

The final buffer chosen for subsequent experiments was the sodium borate/KCl/Tween buffer. This buffer provided the clearest distinction between the positive and negative results (at this tested concentration), with minimal nonspecific nanoparticle aggregation within the negative control, i.e., this buffer provided an overall relatively homogeneous purple to blue color for a positive result and an overall homogeneous (ignoring the nanoparticle gel portion) bright pink color for the negative result. Importantly at this proof-of-concept stage, buffer optimization has not yet been performed. We acknowledge that different buffering conditions may be better suited for our platform. However, we note that buffer optimization studies would require testing with a broad range of target concentrations, reaction times, buffer concentrations, reagent ratios, etc. Buffer performance may be assessed based on platform rapidity, sensitivity, specificity, and ease of distinction based on naked-eye differentiation between positive and negative samples. Moreover, the “ideal” choice of buffer may be subjective because one buffer may provide a better sensitivity, for example; however, it may not yield the optimum distinction (between positive and negative targets) for ease of naked-eye readability. Full optimization of these parameters is beyond the scope of this current proof-of-concept testing stage. Rather, the platform was only optimized in terms of speed and visual distinction between positive and negative samples at this stage. We determined that a combination of 100 μL of vaginal fluid, 5 μL of nanoparticle gel solution, and 45 μL of running buffer were ideal for use with 1.5 cm × 1.5 cm sample pads. This buffer volume was not so large that it diluted the colorimetric results or overflowed the sample pads, and it was not so small that it could not release the nanoparticles from the gel.

### 3.3. Substrate Selection

**Figure 4:**
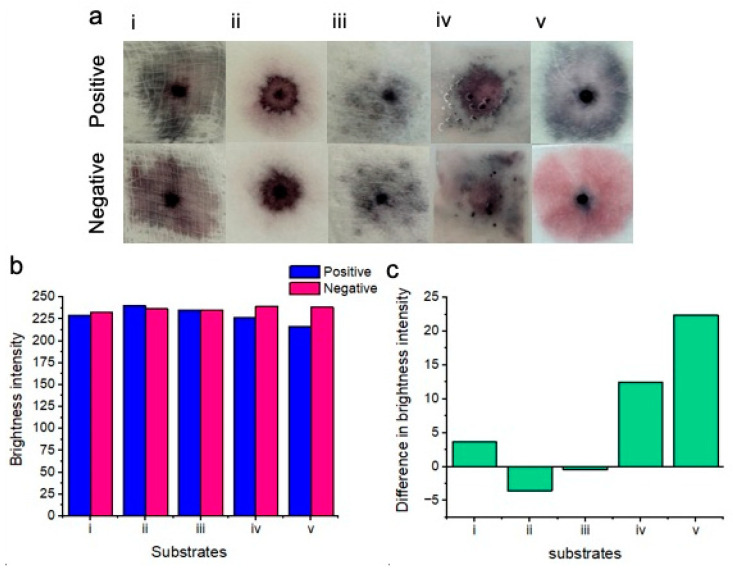
Results of colorimetric naked-eye substrate testing (**a**). Positive results (top) are compared to negative results (bottom) for each of the substrates. Gauze (i) shows no clear distinction between positive and negative samples. Cellulose fiber (ii), sanitary pad (iii), and chromatography paper (iv) positive and negative results are also indistinguishable. Glass fiber (v) shows the best distinction between positive (blue) and negative (pink) results. (**b**) Image processing for red channel brightness intensity for positive and negative samples for each of the substrates is shown. (**c**) The difference in red channel brightness intensity between positive and negative samples for each of the substrates is shown. Notably, the glass fiber substrate (v) showed the best distinction between positive and negative results due to the highest difference in the red channel brightness intensity level.

The choice of substrate was crucial for the success of the platform at this stage of testing (Figure 4). The final substrate was chosen for all future experiments based on its ability to showcase visually distinctive positive results compared to the negative results. Five substrates were tested, including gauze swabs, cellulose fiber sample pads, sanitary pads, chromatography paper, and glass fiber pads. These substrates were selected due to availability, as well as being low-cost, absorbent (for vaginal fluid samples), lightweight, cheap, flexible, and potentially wearable. A concentration of 7.0 × 10^7^ *Candida albicans* cells per ml was tested to ensure the best distinction could be made between positive and negative tests for each tested substrate. The results of the substrate testing describing the clarity of the naked-eye distinction between positive and negative samples are as follows:

Gauze (i): There was no clear distinction between positive and negative samples. Positive samples turned dark purple upon nanoparticle aggregation, whereas negative samples turned a similar color.

Cellulose fiber (ii): There was no distinction between positive and negative samples. Both samples turned purple on this substrate. We believe that this was possibly due to nanoparticles drying out within this substrate.

Sanitary pad (iii): There was no distinction between positive and negative samples when testing with sanitary pad substrates. Significant non-specific nanoparticle aggregation was exhibited between both positive and negative samples. We believe that this may be attributed to significant drying of the nanoparticle solutions.

Chromatography paper (iv): There was no distinction between positive and negative samples when testing on chromatography paper. Upon this substrate, the nanoparticle gel became disrupted, possibly due to the buffer solution spreading the nanoparticles across the surface of the substrate rather than within the substrate.

Glass fiber (v): There was a very clear distinction between positive and negative results when testing on the glass fiber substrate. Positive samples showed significant nanoparticle aggregation and turned dark blue, whereas negative samples remained a bright pink color and free of visible aggregation.

Image processing for red channel brightness intensity for each of the substrates is shown in Figure 4b. Gauze (i), chromatography paper (iv), and glass fiber (v) showed an increase in red channel brightness intensity for the negative results in comparison to the positive results. However, cellulose fiber (ii) and sanitary pad substrates (iii) unexpectedly showed less red channel brightness intensity for negative results when compared to positive results. Image processing showed that the greatest difference in red channel brightness intensity between positive and negative results was achieved with the glass fiber substrate. Consequently, glass fiber was selected for all subsequent experiments due to its performance in differentiating positive “blue” tests from negative “pink” tests. Figure 4c shows the difference in brightness intensity (after image processing) between positive and negative samples for each of the substrates. Negative values for (ii) and (iii) indicate decreased red channel brightness intensity for the negative samples.

### 3.4. Semiquantitative Candida Yeast Cell Detection

**Figure 5:**
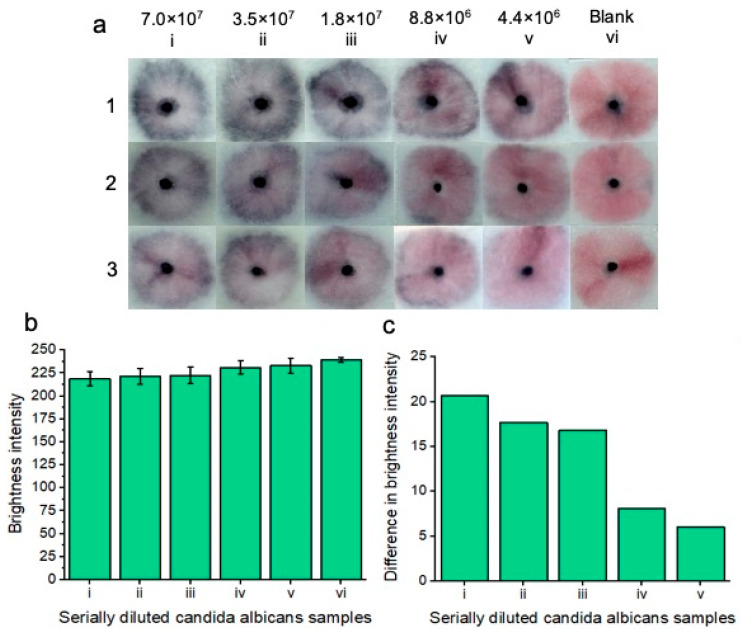
Colorimetric naked-eye results indicate positive tests for five concentrations (7.0 × 10^7^ i), (3.5 × 10^7^ ii), (1.8 × 10^7^ iii), (8.8 × 10^6^ iv), and (4.4 × 10^6^ v) cells per ml of serially diluted *Candida albicans* cells that are spiked into vaginal fluid simulant solution (**a**). Positive “blue” results are compared to the “pink” blank solution (vi). As the concentration of *Candida albicans* cells increases, so does nanoparticle aggregation, and therefore, the sample pads turn increasingly “blue”. Three replicates (1, 2, and 3) show similar results across the respective concentrations, indicating assay reproducibility. All tests are shown after 20 min of development time after adding the running buffer. (**b**) shows image processing results based on red channel brightness intensity for three individual replicates (with error bars), each containing five tested concentrations of *Candida albicans* cells (from (**a**)) in reference to the blank “pink” solution (vi). The difference in red channel brightness intensity between each of the tested concentrations compared to the blank sample is shown in (**c**). Notably, the highest concentration of *Candida albicans* cells (i) differed the most from the blank solution in terms of red channel brightness intensity. As the concentration of *Candida albicans* cells decreased, so did the difference in red channel brightness intensity for each of the samples compared to the blank sample.

To demonstrate a proof-of-concept for *C. albicans* detection, the platform was tested with five different concentrations of *C. albicans* cells across three replicates (Figure 5a). The sodium borate/KCl/Tween-20 buffer combination was chosen for all following experiments due to its superior performance in initial testing. Initially, a stock solution of 7.0 × 10^7^ *C. albicans* cells per ml (in vaginal fluid simulant solution) was created via optical density (OD_600_) analysis.

Then, the stock solution was diluted (1:1) in vaginal fluid simulant solution to an eventual concentration of 4.4 × 10^6^ *C. albicans* cells per ml. Each of the concentrations was compared to the blank solution comprised of vaginal fluid simulant solution only. The results are displayed after 20 min. 20 min was deemed the optimal time to visualize all the colorimetric results at lower concentrations; however, at higher concentrations, a result starts to appear within 5 min. Notably, upon optimization of manual buffer addition to the nanoparticle gel, we believe that our results will reflect indistinguishable replicates. Increasing the concentration of *C. albicans* cells led to increased nanoparticle aggregation and, therefore, an increase in the intensity of the observed “blue” color. All five concentrations of *C. albicans* cells differed in color compared to the blank solution that remained pink. The results indicated that the platform was able to detect *C. albicans* cells within vaginal fluid simulation after 20 min.

Image processing was performed for each of the concentrations and replicates (Figure 5b), and red channel brightness intensity is shown. As the concentration of *Candida albicans* fungal cells decreased, there was an increase in red channel brightness intensity due to decreased nanoparticle aggregation. Figure 5c shows the difference in red channel brightness intensity for each of the concentrations in comparison to the blank test. The higher the concentration of *Candida albicans* cells, the greater the difference in red channel brightness intensity is from the blank solution. The results of Figure 5c represent an average of each of the individual concentrations from Figure 5b. We note that manual handling effects can impact the results. i.e., if careful handling is not given to adding the running buffer dropwise to the nanoparticle gel, the particles may be unevenly displaced across the sample pad, showing an uneven nanoparticle aggregation pattern for positive tests. Notably, once the result has developed, it is also visible for at least two months after testing, i.e., once the platform has been allowed 20 min of development time, the colorimetric result is “locked in”, and this does not significantly change in brightness intensity for the tested *Candida albicans* concentrations or the blank sample. For this proof-of-concept testing, we note that colorimetric naked eye detection is subjective. i.e., a result that may appear “positive” to one person may be perceived as “negative” to another person. As such, we chose to ignore reporting on a limit of detection here, instead opting to show the lowest concentration of tested *Candida albicans* cells that may still be perceived as a “positive” result in reference to the negative blank results.

### 3.5. Nanoparticle Specificity and Dry Sample Testing

**Figure 6:**
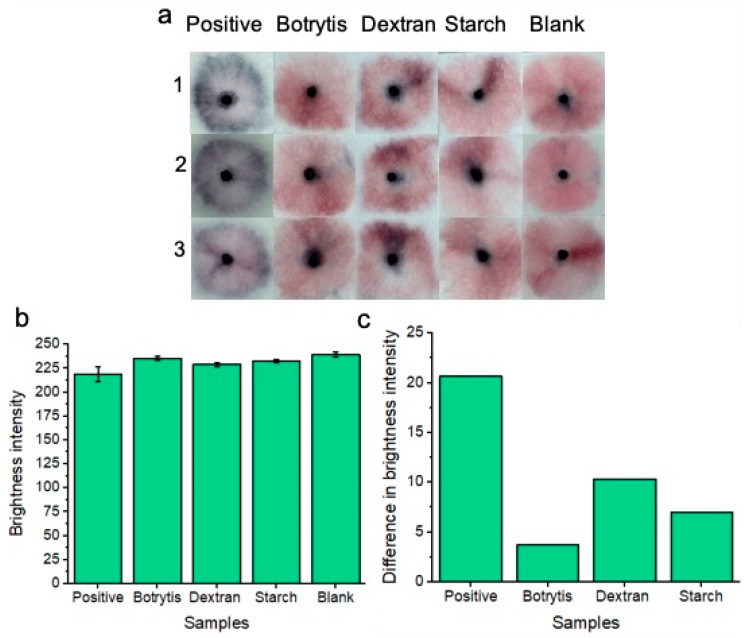
Colorimetric naked-eye results are shown to demonstrate platform specificity. (**a**) Positive “blue” results are compared to negative “pink” results consisting of *Botrytis*, starch, dextran, and blank tests. Three individual replicates (1, 2, and 3) are shown for each test. (**b**) Shows image processing results based on red channel brightness intensity for three individual replicates (with error bars) for each of the tested samples. The difference in red channel brightness intensity between each of the tested samples compared to the blank sample is shown in (**c**). Notably, the positive tests show the greatest difference in red channel brightness intensity from the blank solution when compared to *Botrytis*, dextran, and starch.

Nanoparticle specificity testing (Figure 6a) was conducted following our previous work [16] by using other available glucan-containing substances such as dextran ([C_6_H_10_O_5_]_n_), starch ([C_6_H_10_O_5_]_n_) and the *Botrytis Cinerea* fungus. For this purpose, our aptamer-conjugated gold nanoparticles were expected to aggregate only upon specific recognition of *Candida albicans* glucan molecules. A concentration of 7.0 × 10^7^ *Candida albicans* cells per ml was tested to ensure the best distinction could be made between positive and negative tests for each specificity test. Both Dextran and starch were diluted in a vaginal fluid simulant solution to give respective concentrations of 50 mg/mL. This was to ensure a high concentration of glucan molecules for each of the reagents. At concentrations exceeding 50 mg/mL, solubility was an issue that would unfairly challenge our platform due to matrix effects aiding in nanoparticle aggregation rather than glucan composition alone. As per our prior work [16], an excess of *Botrytis cinerea* mycelium (50 mg) was harvested and was sonicated in addition to samples of vaginal fluid simulant solutions for assay specificity testing. Selecting *Botrytis cinerea* was useful for testing the specificity of our AD1 aptamer-nanoparticle conjugates for two reasons: Firstly, the fungal mycelium provides a significant biological challenge for nanoparticles in solution as nanoparticles may non-specifically aggregate upon the fungal mass. And secondly, it is crucial to test the AD1 aptamer specificity within other glucan molecules as this aptamer is designed to recognize the specific microenvironment of *Candida albicans* BDG molecules only.

100 μL of each respective solution (containing starch, dextran, or *Botrytis*) was used per test on our sample pads. Three individual replicates (1, 2, and 3) were conducted to show reproducibility of the results. Results are shown after 20 min of development time. Each of the non-specific glucan-containing species (dextran, starch, and *Botrytis*) showed a similar “pink” result compared to the blank samples. Image processing of red channel brightness intensity is shown in Figure 6b (with error bars) for three individual replicates of each experiment. Figure 6c shows the difference in red channel brightness intensity between each of the tested samples compared to the blank sample. The results represent the average of the three replicates for each experiment from Figure 6b. The positive “blue” tests differ significantly from the blank “pink” negative test in terms of red channel brightness intensity. *Botrytis*, dextran, and starch each show a minimal difference in red channel brightness intensity from the blank samples. Therefore, the nanoparticles did not significantly non-specifically aggregate under these specificity testing conditions. This implies that our platform is highly specific for the detection of *C. albicans* BDG molecules only.

**Figure 7:**
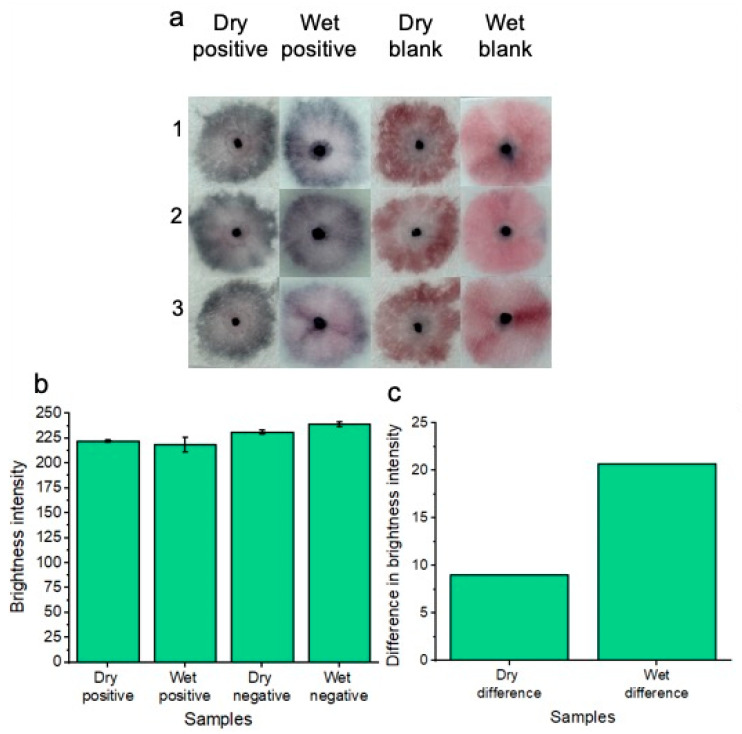
Colorimetric naked-eye results are shown to demonstrate the platform’s ability to test for *Candida albicans* from both wet and dry samples of vaginal fluid (**a**). Three individual replicates are shown for each test. Dry positive tests are visually distinct from dry blank tests, much like the distinction between positive and negative tests of the wet samples. (**b**) shows image processing results based on red channel brightness intensity for three individual replicates (with error bars) for each of the tested samples. The difference in red channel brightness intensity for both the dry and wet positive samples is illustrated compared to the respective blank samples of each testing (wet or dry) condition (**c**). Testing under wet sample conditions gives the best distinction between positive and negative samples; however, a distinction between positive and negative samples can still be made when testing dry samples.

In addition to testing the platform with freshly supplied (wet) *Candida albicans* fungal samples in vaginal fluid simulant solution, dried samples were also tested (Figure 7a). As part of the drying process, samples were added to the pads and were allowed to dry for 1.5 h at 37 °C before nanoparticle gel addition and subsequent running buffer addition. This dry testing was important to assess, given that within a commercial platform, patients would not always be testing on fresh and wet samples of vaginal fluid. i.e., the patient’s vaginal fluid secretions may flow onto the substrate, but the patient may not decide to test the wearable for *Candida albicans* until time has elapsed throughout the day. Three replicates (1, 2 and 3) are shown for the concentration of 7.0 × 10^7^ *Candida albicans* cells per ml in reference to the blank solution. A concentration of 7.0 × 10^7^ *Candida albicans* cells per ml was tested to ensure the best distinction could be made between positive and negative tests across the dry and wet sample conditions. Results are shown after 20 min of development time. The positive samples showed a distinct “blue” color in reference to the “pink” blank tests. Results indicated that the platform is feasible under dry testing conditions for detecting 7.0 × 10^7^ *Candida albicans* cells per ml. However, for future work, other lower concentrations of *Candida albicans* cells will be tested across different drying times to simulate times throughout the day that a patient may decide to test after removing the wearable sample pads. For comparison, the previous results of wet sample testing are included. Image processing for measuring red channel brightness intensity was also conducted (Figure 7b). Three individual replicates are shown with error bars. The results of Figure 7c show a comparison between dry and wet sample positive tests compared to their respective blank samples. The results indicate that testing under wet sample conditions gives the best distinction between positive and negative samples; however, a distinction between positive and negative samples can still be made when testing dry samples.

## 4. Platform Stability Testing

**Figure 8:**
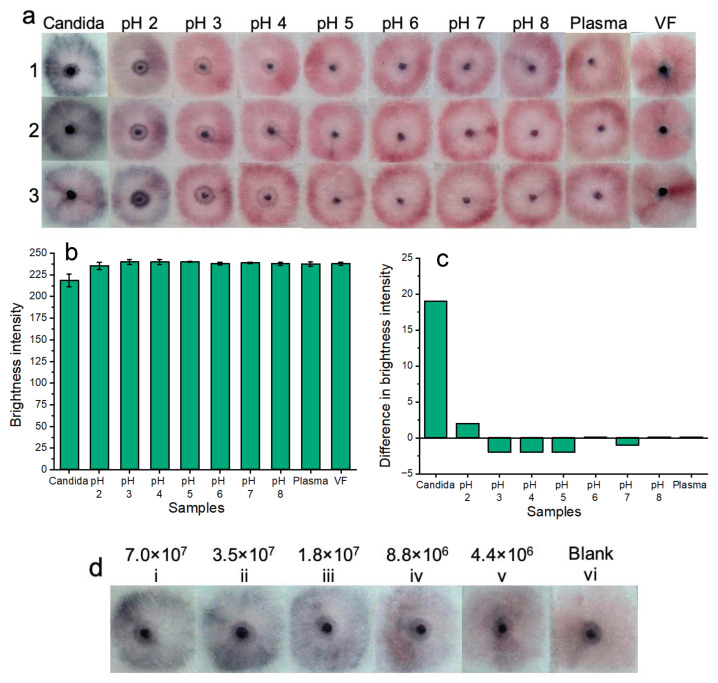
Colorimetric naked-eye results are shown across three replicates (1, 2, and 3) (**a**) to demonstrate platform stability across a wide pH range and within a complex biological setting (plasma). Testing within plasma and a pH range of 3.0–8.0 showed no nonspecific nanoparticle aggregation when compared to blank samples or positive samples containing *Candida albicans*. At pH 2.0, slight nanoparticle aggregation was observed. Positive tests for *Candida albicans* were visually distinct “dark blue” from all other samples (all pH tests, plasma, and blank vaginal fluid samples). (**b**) Shows image processing results based on red channel brightness intensity for three individual replicates (with error bars) for each of the tested samples. (**c**) Shows that positive tests for *Candida albicans* show a large difference in red channel brightness intensity when compared to the blank sample (VF from Figure 8a). The results of pH testing and plasma testing show that all other samples that do not contain *Candida albicans* closely resemble the blank samples in terms of red channel brightness intensity. (**d**) Shows that the colorimetric results of testing the platform with serially diluted samples of *Candida albicans* fungal cells are visible by the naked eye for over one month. However, at lower concentrations of fungal cells, these results are not as bright as testing with fresh samples of *Candida albicans*.

To assess platform stability (Figure 8a), nanoparticle gel solutions were tested under different pH conditions, as well as within a complex biological environment (human plasma). Nanoparticle gel solutions were deemed to be robust and stable if they did not nonspecifically aggregate (turn dark blue or purple) under the tested conditions.

For pH testing: solutions of vaginal fluid simulant (ranging from pH 2.0 to 8.0) were added to sample pads, followed by 5 μL of nanoparticle gel solution and 45 μL of buffer solution. The results were allowed to develop for 20 min before being recorded. The results of three experimental replicates (1, 2, and 3) are illustrated in Figure 8. This range was important to test given that normal vaginal pH ranges from 3.8 to 5.0, but vaginal pH may be as high as 7.5 during vaginal infection [33]. If a patient suffers from coinfection, ensuring nanoparticle stability over this wide pH range eliminates potential false-positive results that may occur due to nanoparticle pH-induced instability alone. The results indicated that nanoparticle gel solutions remained free of aggregation and were stable between the pH range of 3.0 to 8.0. At pH 2.0, nanoparticles showed aggregation by turning blue/purple. Each of the pH-based results was shown in reference to the blank solution containing the standard biologically relevant and viscous vaginal fluid simulant solution (pH 4.2) used throughout all prior experiments. Additionally, the pH-based results were also compared to the results of a positive sample for *Candida albicans* (7.0 × 10^7^ cells/mL). Notably, positive results for *Candida albicans* are visually distinct from samples that do not contain *Candida albicans* at a pH range between 2.0 and 8.0. The results of pH testing indicate potential suitability for future clinical application due to nanoparticle gel solution stability across a wide vaginal fluid pH range.

For complex biological media testing: undiluted human plasma samples were used in place of vaginal fluid simulant samples. Plasma was used due to laboratory availability and its biological composition, which may challenge our platform by inducing nonspecific nanoparticle aggregation. A volume of 100 μL of plasma was added to the sample pads, followed by 5 μL of nanoparticle gel solution and subsequent addition of 45 μL of buffer solution. Three experimental replicates were performed, and the “yellow” color of the plasma is evident in each test. The results of the plasma tests indicate potential suitability for future clinical application due to the stability of the nanoparticle gel solutions within complex biological fluids. i.e., when nanoparticle gel solutions were tested within plasma samples, no non-specific aggregation occurred. For reference, positive tests containing *Candida albicans* spiked into vaginal fluid simulant solution were distinctly “dark blue”, and tests containing plasma without *Candida albicans* were distinctly “pink”, resembling the blank solution.

Additionally, we noted that there was a “ring” shaped pattern that is present across all pH tests as well as plasma sample testing when compared to blank (VF) solutions. We believe that for the pH samples, this may be due to the lower viscosity of the vaginal fluid simulant solution, which may have more easily flowed toward the edges of the sample pads. In the more viscous negative VF samples, we believe that the nanoparticles are more dispersed across the viscous VF solution and, therefore, cause a larger area of dispersed pink and unaggregated nanoparticles. In the case of the plasma samples, the “ring” pattern may be caused by the liquid buffer transporting the unaggregated nanoparticles towards the sample pad edges, as there was no viscous and homogeneous vaginal fluid simulant solution present to uniformly suspend the nanoparticles. With a longer development time, we believe that these “ring” patterns will become less visible and more homogenous across the sample pads.

Red channel brightness intensity analysis (Figure 8b) was performed for three replicates (with error bars) of each of the colorimetric samples of Figure 8a. Figure 8c shows the difference in red channel brightness intensity between each of the tested samples compared to the blank sample (Figure 8a (VF)). The results support the colorimetric results of Figure 8a, i.e., tests across pH 3.0 to 8.0, as well as tests for plasma samples, resembled the blank sample (VF) in terms of similar red channel brightness intensity. The results of pH 2.0 sample testing showed a slightly decreased red channel brightness intensity when compared to the blank samples (VF). The positive *Candida albicans* tests showed the lowest levels of red channel brightness intensity due to the aggregated, dark blue nanoparticles. The results of Figure 8b demonstrate that there is a large difference in red channel brightness intensity between positive samples containing *Candida albicans* compared to the blank sample (VF) red channel brightness intensity. Red channel brightness intensity levels ranging from pH 2.0 to 8.0, as well as plasma samples, only show a tiny difference in red channel brightness intensity compared to the blank sample. Results for pH levels 3.0, 4.0, 5.0, and 7.0 show negative values because these had a slightly higher red channel brightness intensity when compared to the blank solution. This result is not significant as the “ideal” maximum value of red channel brightness intensity that indicates a negative result is yet to be optimized.

We believe that optimization and further investigation need to be performed to confirm the cause of nanoparticle aggregation within the middle gel portion of the sample pads of negative tests. We believe that during certain buffering conditions, some nanoparticles may remain trapped within the porous matrix of the gel, which may cause unwanted nanoparticle interactions. Additionally, the middle portion of the pads may experience drying under certain buffering/detergent/salt conditions, which may cause unwanted non-specific nanoparticle aggregation.

We believe that the composition of the running buffer and its interaction with the nanoparticles/nanoparticle gel plays a significant role in this aggregation phenomenon observed in the middle of the sample pads. Nevertheless, we may consider optimizing previously investigated buffer combinations to eliminate this nonspecific middle nanoparticle aggregation. However, there may be a tradeoff in terms of sensitivity or vibrancy of colorimetric results. However, at this stage of testing, the slight unexplained aggregation of the nanoparticle gel portions did not appear to affect the overall colorimetric results of our platform. I.e. for all negative tests, a bright pink/red color was still observed.

Notably, while the results are subjective, the colorimetric results are visible on the sample pads after even one month (Figure 8d), and the colorimetric result is “locked in” as opposed to a solution-based result that may destabilize and aggregate over time.

## 5. Conclusions

*Candida albicans* is a commensal fungus that exists within the bodies of most healthy people without causing any symptoms. However, *Candida albicans* is an opportunistic fungus that will become pathogenic, harmful, and potentially deadly if biological conditions permit. This is often the case for immunocompromised patients. Detection of fungal pathogens such as *Candida albicans* currently requires access to trained medical staff and laboratories. This process is slow, inconvenient, costly, and often embarrassing for the patient. This may cause the patient to self-diagnose (often wrongfully) and to self-treat with over-the-counter antifungal treatments, which can cause eventual antifungal resistance. Therefore, there is a special need for a discrete, sensitive, specific, cost-effective, and rapid platform that women can use within the comfort of their homes for *C. albicans* detection. To achieve this goal, we created a wearable platform for *Candida albicans* detection based on aptamer recognition of *Candida albicans* β-1,3-D-glucans. Our platform utilizes thiolated aptamer-conjugated gold nanoparticles that are underpinned by surface plasmon resonance properties. In the presence of *Candida albicans*, a redshift in the UV-visible absorbance occurs based on nanoparticle aggregation that is proportional to the amount of *Candida albicans* β-1,3-D-glucans. This reveals a blue color that is detectable by the naked eye. In the absence of *Candida albicans*, nanoparticles remain free from aggregation, and they remain pink. Our wearable platform includes three steps: (i) collection of vaginal fluid from wearable glass fiber sample pads; (ii) addition of nanoparticle gel and running buffer, and (iii) a colorimetric result based on the presence or absence of *Candida albicans* fungal cells. Combining the nanoparticles with the gel achieved two functions that supported our platform—(i) it provided a way to stabilize the gold nanoparticles, keeping them free from unwanted aggregation, and (ii) it provided a way for easy application onto the sample pads, allowing careful control of particle position and dispensed volume.

To the best of our knowledge, no other colorimetric, wearable gold nanoparticle aggregation-based platform exists for the detection of *Candida albicans*. Within future works, we will improve upon this platform to reduce the error associated with manual handling steps, i.e., we may incorporate the nanoparticles within the sample collection pads; however, extensive analysis will need to be performed to ensure that these tests are safe for patients in case of skin contact. This will be achieved by investigating skin-friendly top layers to protect the skin while allowing vaginal fluid containing *Candida albicans* to still be deposited on the glass fiber pads. We will also thoroughly investigate other buffer combinations and concentrations to improve platform sensitivity and specificity without sacrificing the quality of the naked eye results. In the present proof-of-concept study, we have only optimized for rapidity and color change brightness intensity for the easiest distinction between positive and negative samples. We also acknowledge the need to collaborate with clinicians to tune platform sensitivity to determine a cutoff value for *Candida albicans* cell counts that will differentiate colonization from infection. With some modification, we believe that our platform can be adapted to detect other fungal pathogens from wearable substrates for older people and babies. Furthermore, analysis of red channel brightness intensity shows the potential for incorporating our platform into a camera and software processing-based platform. While this would add to platform costs and required equipment, this would potentially overcome the limitation of colorimetric analysis. Specifically, giving a “yes” or “no” answer based on a predetermined cutoff color corresponding to an exact number of detected *Candida albicans* fungal cells rather than relying on color interpretation by the naked eye. Overall, we believe that this work will contribute to revolutionizing women’s health by giving patients more control over their own health, encouraging them to gain a better understanding of disease rather than relying on clinicians.

## Data Availability

The original contributions presented in the study are included in the article. Further inquiries can be directed to the corresponding author.
